# Detailed protocol for germ-free *Drosophila melanogaster* colonization with *Propionibacterium* spp. biofilms

**DOI:** 10.1016/j.xpro.2022.101342

**Published:** 2022-04-22

**Authors:** Vicky Bronnec, Oleg A. Alexeyev

**Affiliations:** 1 Department of Medical Biosciences/Pathology, Umeå University, 90 187 Umeå, Sweden

**Keywords:** Health Sciences, Microbiology, Microscopy, Model Organisms

## Abstract

In this protocol, we describe a germ-free *Drosophila melanogaster* model to investigate anaerobic bacterial biofilms. We detail how to establish *Propionibacterium* spp. biofilms in the fruit fly’s gut using an easy to carry out method.

For complete details on the use and execution of this protocol, please refer to Bronnec and Alexeyev (2021) and Bronnec et al. (2022).

## Highlights

• Detailed protocol for the generation of germ-free *Drosophila melanogaster*
• Oral infection of germ-free fruit flies and *Propionibacterium* spp. colonization
• Visualization by microscopy of *Propionibacterium* spp. biofilms in fruit flies
• *In vivo* exposed epithelial cells in low oxygen environment

## Graphical abstract

**Figure:**
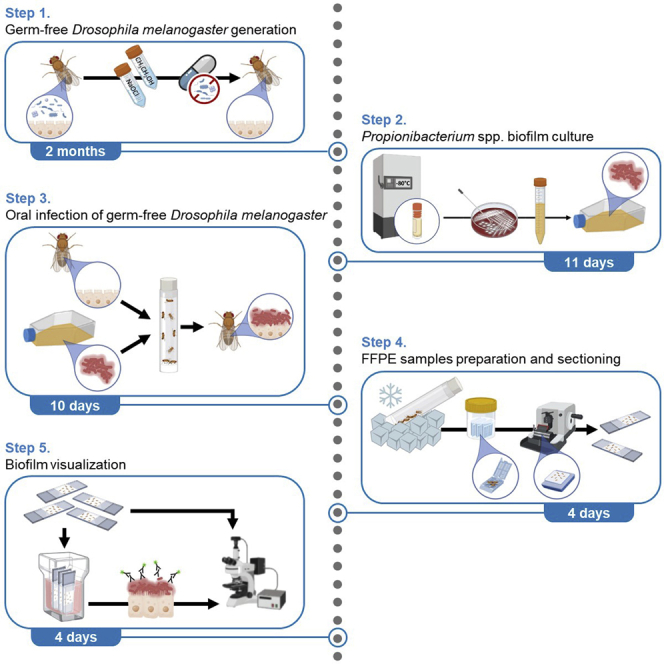

## Before you begin

This protocol describes how to create an *in vivo* biofilm model system in *Drosophila melanogaster* (fruit fly) to study *Propionibacterium* spp. (*Propionibacterium acnes*, *Propionibacterium avidum*, *Propionibacterium granulosum*) mono-species biofilm. *P. acnes*, an anaerobic bacterium, is believed to be an important factor in the pathogenesis of acne vulgaris due to its ability to form biofilms, defined as bacterial aggregates embedded in an extracellular protective matrix (Flemming and Wingender, 2010; Flemming et al., 2016). Acne research has long been plagued by the lack of a suitable *in vivo* model (Bronnec and Alexeyev, 2021). The gastrointestinal tract of fruit flies harbors an epithelium barrier in a low oxygen environment suitable for the colonization by anaerobic bacteria such as *Propionibacterium* spp. and biofilm formation (Bronnec and Alexeyev, 2021).

Feeding fruit flies with *Propionibacterium* spp. leads to the colonization of their digestive tract and the development of a biofilm. The protocol below describes the specific steps for the development and maintenance of a sterile line of *D. melanogaster* and infection with preformed biofilm of *Propionibacterium* spp.

The method is however adjustable to mimic different environmental conditions by modifying the fruit fly diet. We have used our *in vivo* biofilm model system to investigate *Propionibacterium* spp. biofilm in different conditions (Bronnec and Alexeyev, 2021; Bronnec et al., 2022). In those publications, the fruit fly food was supplemented with a lipid solution. Development of a biofilm in lipid-rich environment in the fruit fly gut (low level of oxygen) mimics to some extent the natural condition encountered in the pilosebaceous unit in the skin. Fruit flies infected with *Propionibacterium* spp. biofilm were also orally treated with biofilm dispersing enzymes to evaluate their biofilm degrading activity *in vivo* (Bronnec and Alexeyev, 2021; Bronnec et al., 2022). These alternatives are not reported in this protocol. Herein, we describe visualization methods to evaluate the biofilm with light and fluorescent microscopy. Bright-field observations allow quantification of biofilms without computational processing of whole fly tissue sections. Immunolabeling assays provide a specific detection of *Propionibacterium* spp. and allow the visualization of the biofilm as well as its organization in the gut. Scanning Electron Microscopy (not described in this protocol) allows investigation of the biofilm matrix architecture (Bronnec and Alexeyev, 2021).

Before starting the experiment, a few preliminary steps must be taken. Good laboratory practices should be followed in order to perform experiments in a sterile and safe way for the user and to preserve non-contaminated biological materials. A general information about the sterilization process is provided. To generate germ-free (GF) fruit flies, we recommend setting-up a sterile in-house disposable washing kit which contains all the material necessary to wash fruit flies or their eggs. Procedures for maintenance of sterility during fruit flies transfer and anesthesia are provided in detail. Animals were reared at 25°C and 60% humidity and a note is provided regarding the fruit fly disposal.

1. Standard microbiological practices.
  All procedures involving the fruit fly and bacteria manipulations should be performed in sterile conditions following usual good microbiological laboratory practices (Chauhan and Jindal, 2020; Siddiquee, 2017). Bacterial cultivation can be performed using a Bunsen burner but fruit flies’ manipulations must be performed in a biological safety cabinet (BSC).
  In order to minimize the risk of contamination while using the BSC the user should follow the general guidelines hereafter:
  a. Purge the BSC by turning it “ON” at least 5 min before starting the work.
  b. Disinfect the cabinet with a bleach solution or 70% ethanol.
  c. Wipe every item introduced in the BSC with 70% ethanol.
  d. Wear gloves and disinfect them with 70% ethanol before starting and when manipulating non-sterile materials.
  e. Organize the work zone in the BSC with “clean”, “working” and “dirty” area to avoid cross-contaminations.
  f. Minimize movements and do not block the airflow grill to not disturb the air flow and compromise the sterility.
    ***Note:*** These guidelines aim at presenting the main steps in order to work in aseptic conditions to protect the experiment from contaminations. Personal protective laboratory clothing must be worn as well as good microbiological practices should be followed to protect the user (Chauhan and Jindal, 2020; Siddiquee, 2017).
2. Sterilizations.

Several sections of this protocol require sterile materials and media. Single-use disposable materials are purchased sterile. Some liquid solutions (antibiotics, glucose and sucrose) are sterilized in aseptic conditions by filtration with a 0,2 μm filter. For other materials and media, sterilization process is performed using a bench top autoclave with a cycle of 20 min at 121°C.

**Figure 1:**
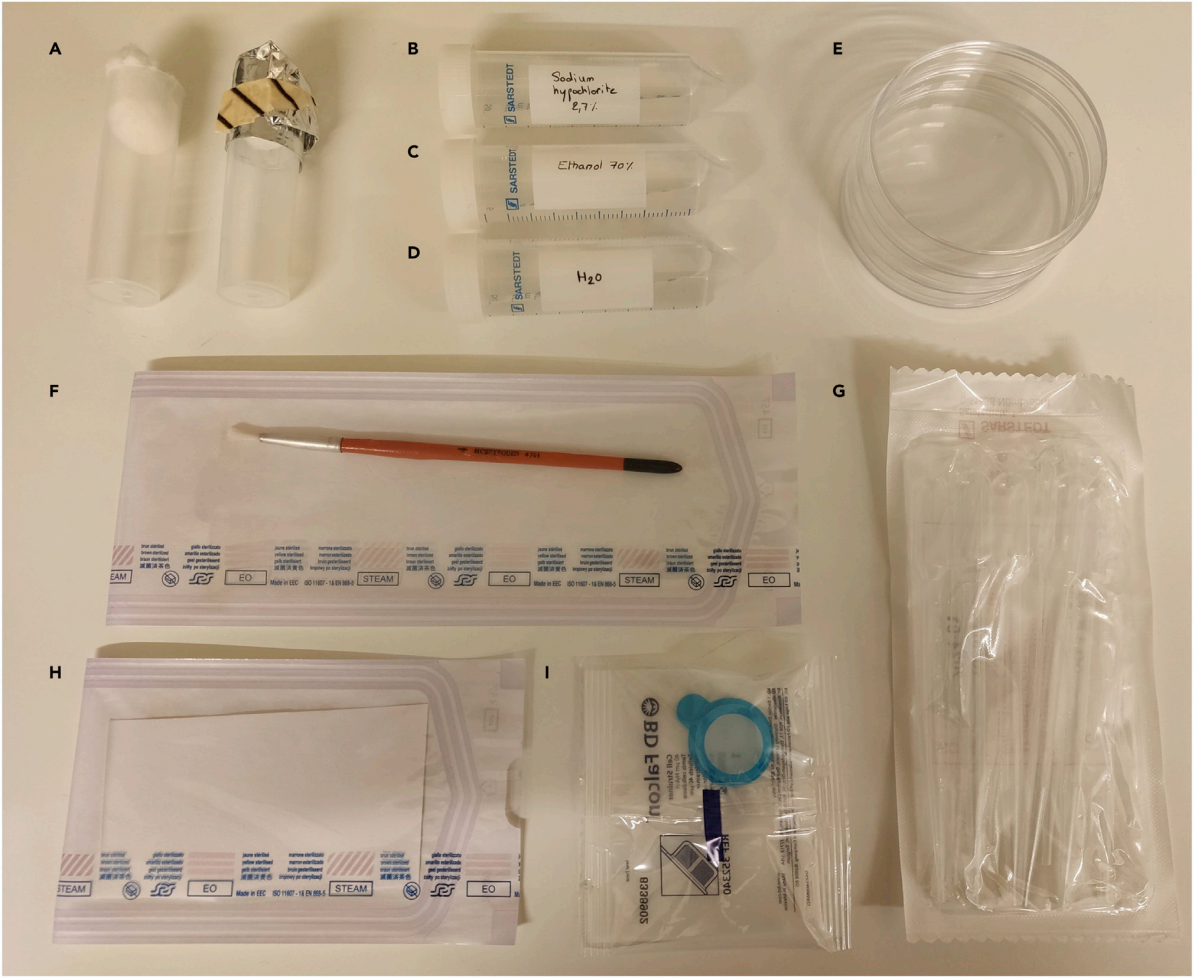
Disposable washing kit to generate germ-free *Drosophila melanogaster* (A) Fruit fly vials should be closed with a cotton plug and tightly wrap in aluminum foil for autoclaving. (B–D) Sodium hypochlorite and ethanol are diluted in sterile milli-Q water. All liquid solutions should be heated at 30°C before use. (E, G, and I) Plastic disposables purchased sterile. (F and H) Painting brush (washed in 70% ethanol) and blotting papers (disposable) are sterilized in autoclavable envelopes.

3. Preparation of the washing kits for generation of germ-free *Drosophila melanogaster.*
  This section describes how to prepare in-house disposable washing kits containing the materials necessary to wash fruit flies and eggs to generate GF fruit flies (Figure 1).
  ***Note:*** The user must work under sterile conditions throughout.
  a. Empty vials autoclaved with a cotton plug and tightly wrap in aluminum foil (Figure 1A).
    ***Note:*** When using, vials should be totally dry, without condensation left on the walls.
  b. 2,7% sodium hypochlorite warmed at about 30°C before use (Figure 1B).
  c. Ethanol 70% warmed at about 30°C before use (Figure 1C).
  d. Sterile milli-Q water warmed at about 30°C before use (Figure 1D).
  e. Sterile Petri dish (Figure 1E).
  f. Sterile painting brush (Figure 1F).
  g. Sterile plastic transfer pipette (Figure 1G).
  h. Sterile blotting paper (Figure 1H).
  i. Sterile 40 μm cell strainer (Figure 1I).
    ***Note:*** Petri dishes, transfer pipettes and cell strainers are purchased sterile. Water, painting brush and blotting papers are sterilized in autoclave (Figure 1).

4. Fruit flies transfer between vials.
  The following steps describe how to transfer fruit flies between vials after a stun or anesthesia. Commonly, in fruit fly labs, CO_2_ guns are introduced in the vial to anesthetize them. In the present protocol, we suggest two other methods in order not to compromise sterility.
  For routine and quick transfers onto fresh food a stun is sufficient and highly recommended during infection steps. For other transfers with a population counting or washing, an anesthesia on ice is necessary.
  ***Note:*** The user must work under sterile conditions throughout.
  **CRITICAL:** Fruit flies transfer should be performed in BSC. Standard practices and procedures should be followed to avoid contaminations.
  a. Spray-off with 70% ethanol all the fruit fly vials including the outside of the cotton plug before starting. Let it dry before continuing with the next step.
  b. To stun the fruit flies, gently tap the vial down on the surface until all of the fruit flies fall to the bottom. Fruit flies will be immobilized for a few seconds (± 2 s). Maintain them at the bottom by continuously taping the vial down.
    ***Note:*** As soon as the vial is not tapped down, fruit flies start moving and climb on the wall of the vial. The user should be quick and practice fly handling.
  c. As an alternative, place the vial horizontally on ice for few minutes (rotate it from time to time to chill the vial wall homogeneously). Fruit flies will stop moving and “fall asleep” within a few minutes, the longer the fruit flies are on ice, the longer they will be “asleep”.
    ***Note:*** Too much time on ice will kill the fruit flies.
  d. To transfer the fruit flies, prepare the two vials close to each other and open the new vial.
  e. Tape down the vial with the fruit flies.
  f. Quickly remove the cotton plug and invert it on the new one.
  g. Tap the vials down together to transfer all fruit flies.
    ***Note:*** If fruit flies are just stunned, continue to tap down to immobilize the fruit flies at the bottom while closing the new vial.
  h. Place vials sideways to let the fruit flies recover.
    ***Note:*** If the media is too moist / liquid, the fruit flies will get stuck in it and die (troubleshooting 1).
    **CRITICAL:** Sterile manipulation of fruit flies is performed in a BSC. The transfer between vials should be as quick as possible and fruit flies should be maintained immobilized or anesthetized at the bottom of the vial otherwise the air flow in the cabinet will aspirate the fruit flies.
    **CRITICAL:** During population counting (to maintain a certain density in vials), fruit flies should be totally under anesthesia. As soon as they start to wake-up they deploy their wings and are more easily aspirated by the BSC flow.

5. Fruit flies incubation.

The optimal environment for *D. melanogaster* is an incubation at 25°C with about 60% humidity. If no incubator is available fruit flies can be incubated between 18°C and 25°C out of direct sunlight.

***Note:*** A dry environment should be avoided to maintain the food moist and reduce the frequency between vial transfer.

***Note:*** During maintenance, fruit flies should be transferred approximately every 2–3 weeks depending on the population density. This duration time should be determined empirically according to the culturing and incubation conditions. Visual observation of the quality and quantity of the rearing media in the tubes should be performed.

***Note:*** Population density for fruit fly crossing is about 50 fruit flies per vial. To maintain a low rate of crossing the user can reduce the number of fruit flies per vials (< 10). For each experiment a population of approximately 30 fruit flies is used and they are not sorted according to their gender.

6. Fruit flies disposal.

During experiments, some fruit flies have to be discarded. The user can euthanize fruit flies by freezing them (in a vial) for at least 24 h or by dumping stunned fruit flies in a container (bottle or beaker) filled with ethanol or isopropanol. Insect waste can then be disposed of in the same manner as other solid, non-sharps biohazardous waste following the waste management rules of the user’s laboratory.

## Key resources table

**Table:** 

REAGENT or RESOURCE	SOURCE	IDENTIFIER
**Antibodies**		

Mouse monoclonal # 8313, anti-*Propionibacterium acnes; used undiluted*	Agrisera	N/A
Chicken polyclonal IgY1, anti- *Propionibacterium granulosum; used diluted at 1/400 in* 2,5% Normal Horse Serum Blocking Solution	Agrisera	N/A
Rabbit polyclonal Timby, anti- *Propionibacterium avidum; used diluted at 1/10,000 in* 2,5% Normal Horse Serum Blocking Solution	Agrisera	N/A
Goat anti-Mouse IgG (H+L), Superclonal™ Recombinant Secondary Antibody, Alexa Fluor 555*; used diluted at 1/500 in* 2,5% Normal Horse Serum Blocking Solution	Thermo Fisher Scientific, Invitrogen	Cat#A-28180
Goat anti-Mouse IgG (H+L) Cross-Adsorbed Secondary Antibody, Alexa Fluor 488*; used diluted at 1/500 in* 2,5% Normal Horse Serum Blocking Solution	Thermo Fisher Scientific, Invitrogen	Cat#A-11001
Donkey anti-Rabbit IgG (H+L) Highly Cross-Adsorbed Secondary Antibody, Alexa Fluor 555*; used diluted at 1/500 in* 2,5% Normal Horse Serum Blocking Solution	Thermo Fisher Scientific, Invitrogen	Cat#A-31572
Alexa Fluor® 647 AffiniPure F(ab')₂ Fragment Donkey Anti-Chicken IgY (IgG) (H+L)*; used diluted at 1/500 in* 2,5% Normal Horse Serum Blocking Solution	Jackson ImmunoResearch Europe Ltd.	Cat#703-606-155

**Bacterial and virus strains**		

*Propionibacterium acnes* (*Cutibacterium acnes*) strain KPA171202	DSMZ	Cat#DSM 16379
*Propionibacterium granulosum* (*Cutibacteriumgranulosum*) strain ATCC 25564	DSMZ	Cat#DSM 20700
*Propionibacterium avidum* (*Cutibacteriumavidum)* strain ATCC 25577	DSMZ	Cat#DSM 4901

**Chemicals, peptides, and recombinant proteins**		

Ethanol	VWR	Cat#20823.362
2,5% Normal Horse Serum Blocking Solution	Vector Laboratories	Cat#S-2012
4-Hydroxybenzoic acid	Sigma-Aldrich	Cat#W398608
Agar	Fisher Scientific	Cat#10572775
Ampicillin	Sigma-Aldrich	Cat#59349
Brain Heart Infusion broth	Sigma-Aldrich	Cat#53286
Calcium chloride, CaCl2	Sigma-Aldrich	Cat#C1016
Ciprofloxacin	Sigma-Aldrich	Cat#17850
Citifluor™ Antifadent Mountant Solutions AF1 glycerol-PBS	Citifluor	Cat#17970-25
Columbia Blood Agar Base	Thermo Fisher Scientific	Cat#CM0331B
DAPI (4′,6-diamidino-2-phenylindole)	Sigma-Aldrich	Cat#D9542
EDTA, Ethylenediaminetetraacetic acid	Sigma-Aldrich	Cat#E9884
Erythromycin	Sigma-Aldrich	Cat#E5389
Formalin	Sigma-Aldrich	Cat#HT501128
Glucose	VWR	Cat#101174Y
Horse Blood	Håtunalab	Cat#139
Kanamycin	Sigma-Aldrich	Cat#K1377
Magnesium sulfate, MgSO_4_.6H_2_O	Sigma-Aldrich	Cat#M7506
Peptone	Sigma-Aldrich	Cat#83059
Propionic acid	Sigma-Aldrich	Cat#402907
Proteinase K	Sigma-Aldrich	Cat#P2308
Sodium hypochlorite solution	Sigma-Aldrich	Cat#1056142500
Sucrose	Fisher Scientific	Cat#11482751
Tris base	Sigma-Aldrich	Cat#T1503
Triton X-100	Sigma-Aldrich	Cat#X100
VECTOR “Antigen unmasking solution” Low pH	Vector Laboratories	Cat#H-3300
Xylene, Extra Pure	Fisher Scientific	Cat#11498922
Yeast Brewers	Sigma-Aldrich	Cat#Y4625
Yeast extract	Sigma-Aldrich	Cat#Y1625
Paraffin, Paraplast®	Sigma-Aldrich	Cat#P3558
Biotium CoverGrip™ Coverslip Sealant	Fisher Scientific	Cat#NC0154994
Formalin solution, neutral buffered, 10%	Sigma-Aldrich	Cat#HT501128-4L

**Experimental models: Organisms/strains**		

Wild-type *Drosophila melanogaster* genotype W1118 iso; 2-iso; 3-iso; no gender distinction	Gift from Maria Kim, Department of Molecular Biology, Research group Jan Larsson, Umeå University	N/A

**Software and algorithms**		

Zeiss Zen Blue 3.3	ZEISS	https://www.zeiss.com/microscopy/us/products/microscope-software/zen.html

**Other**		

Glass fiber filter 934 ah 24 mm	VWR	Cat#516-2704
Swingsette™ biopsy cassettes	Simport™ Scientific	Cat#M516-5
Anaerogen 2,5l anaerobic bags	Fisher Scientific	Cat#1026-9582
Autoclaves, VAPOUR-Line Lite	VWR	Cat#481-0846
BD GasPak™ EZ Gas Generating Systems Incubation Containers	Fisher Scientific	Cat#10118924
Cellpath Stainless-steel Reusable Base Molds	Fisher Scientific	Cat#22-222-033
Cover glasses, Menzel Gläse	VWR	Cat#630-1843, 630-1845
EasyDip™ Slide Staining Kit	Simport™ Scientific	Cat#M906-12AS
Eppendorf® Centrifuge 5424/5424R	Sigma-Aldrich	Cat#EP5404000537
Epredia™ HM 355S Automatic Microtome	Fisher Scientific	Cat#23-900-672
Falcon® 40 μm Cell Strainer	Corning	Cat#352340
Feather s35 microtome blade	Histolab Products AB	Cat#4100
Filtropur S 0,2	Sarstedt	Cat#83.1826.001
Fisherbrand™ *Drosophila* Vials	Fisher Scientific	Cat#15820275
Fisherbrand™ Nonsterile Cotton Balls	Fisher Scientific	Cat#22-456-883
Gel blotting sheets, GB003, Whatman	VWR	Cat#732-2760
Grant Digital Waterbath Type Sub Aqua 18 Plus	Grant	Cat#SAP18
ImmEdge Hydrophobic Barrier PAP Pen	Vector Laboratories	Cat#H-4000
Incubator, ECOCELL 22 - ECO line	MMM Medcenter Einrichtungen GmbH	Cat#1.4301
Inoculation loop 10 μL	Sarstedt	Cat#86.1562.010
Integra Biosciences™ Pipetboy acu 2 Pipette Controller	Fisher Scientific	Cat#10798252
Leica Microsystems Immersion Oil for Microscopes	Fisher Scientific	Cat#11944399
Histosette® ii - base only for tissue cassettes in e-z load™ stacks	Simport™ Scientific	Cat#M482
Histosette® ii - lids only for biopsy cassettes in e-z load™ stacks	Simport™ Scientific	Cat#M483
Multi-purpose container, 70 mL, (LxØ): 55 × 44 mm, graduated, PP	Sarstedt	Cat#75.9922.744
ORCA-Flash 4.0 LT digital CMOS camera	Hamamatsu	Cat#C11440-42U30
Oxoid™ AnaeroJar™ Base, Jar	Fisher Scientific	Cat#AG0026A
Petri dish	Sarstedt	Cat#82.1472
SafeSeal reaction tube, 1.5 mL, PP	Sarstedt	Cat#72.706
See-through Heat-sealable Pouches	Steriking, WIPAK	Cat#S7; Cat#S3
Staintray™ 10 slides staining system, base with black lid	Simport™ Scientific	Cat#M918-2
SuperFrost Plus GOLD white Adhesion slide	Fisher Scientific	Cat#11976299
Swann-Morton™ Carbon Steel Sterile Scalpel Blades	Fisher Scientific	Cat#11728363
TC Flask T25, Stand., Vent. Cap	Sarstedt	Cat#83.3910.002
Thermo Scientific™ SuperFrost™ Microscope Slides, Cut (White)	Fisher Scientific	Cat#12134682
Transfer pipette	Sarstedt	Cat#86.1171.001
Tube, 10 mL, (LxØ): 100 × 16 mm, PP, with print	Sarstedt	Cat#62.9924.284
Water bath HIR-3D Round, with lighting & digital display, KUNZ	Histolab Products AB	Cat#10064
Zeiss Axio Imager M2 microscope	Carl Zeiss Vision	Cat#AxioImagerM2

## Materials and equipment

### Columbia blood agar plates

Columbia Blood Agar Base (39 g/L) is prepared according to the manufacturer’s instructions and sterilized by autoclaving. After cooling under 50°C the media is supplemented with 5% v/v of sterile horse blood, well mixed and manually pour into Petri dishes (± 18 mL/dish) in sterile conditions. After solidification, Columbia blood agar plates are stored upside down at 4°C.

### Brain Heart Infusion broth and solid media

The media formulations presented in Table 1 are based on the commercial media Brain Heart Infusion broth prepared according to the manufacturer’s instructions. All media should be prepared in a sterile way.

**Table 1:** Media used in the protocol

Media name	Component (final concentration)	Preparation	Advices and comments
BHI	Brain Heart Infusion broth (37g/L)	Dissolve 9,25 g of BHI powder in 250 mL of distilled water. Sterilize by autoclaving.	Prepared in a 500 mL bottle.
Glucose	Glucose (50 g/L)	Dissolve 10 g of glucose in 200 mL of distilled water. Stir until complete dissolution of the powder^a^. Sterilize by filtration through a 0,2 μm filter.	Sterile aliquots of 11 mL can be stored at −20°C and thawed in a water bath at 37°C when needed.
Sucrose	Sucrose (500 g/L)	Dissolve 50 g of sucrose in 100 mL of distilled water. Stir until complete dissolution of the powder^a^. Sterilize by filtration through a 0,2 μm filter.	The solution is highly concentrated, making it thick and difficult to filter. Sterile aliquot of 11 mL can be stored at −20°C and thawed in a water bath at 37°C when needed.
Agar	Agar (15 g/L)	Dissolve 3 g of agar powder in 200 mL of distilled water. Sterilize by autoclaving.	After sterilization, agar bottles can be stored between 18°C and 25°C. When needed, loosen the cap and melt the agar in a microwave^b^.
BHI_g_	Brain Heart Infusion broth (37 g/L) supplemented with glucose (2 g/L)	Add 10,5 mL of sterile glucose (50 g/L) to 250 mL of sterile BHI. Mix thoroughly.	
BHI_s_	Brain Heart Infusion broth (37 g/L) supplemented with sucrose (100 g/L)	Add 2 mL of sterile sucrose (500 g/L) to 8 mL of sterile BHI_g_. Mix thoroughly.	Sterile aliquot of 1 mL can be stored at −20°C and thawed in a water bath at 37°C when needed.
BHIA_gsy_ (with or without antibiotics)	Brain Heart Infusion broth (37 g/L), agar (15 g/L), yeast extract (60 g/L), supplemented with glucose (5 g/L), sucrose (10 g/L) and antibiotics.	Dissolve BHI powder (1,85 g), agar (0,75 g), yeast extract (3 g) in 35 mL of distilled water. Sterilize by autoclaving. When the solution is prehensible add 5 mL of sterile glucose (50 g/L) and 10 mL of sterile sucrose (500 g/L). Add antibiotics if necessary (Table 3). Stir and distribute 2 mL in sterile fruit fly vials before solidification.	Preparation in a 100 mL bottle. We recommend using a magnetic stir bar for homogenization and to keep it in the bottle for autoclaving^b^. After sterilization, bottles of BHI / agar / yeast extract can be stored between 18°C and 25°C. Before adding glucose and sucrose, warm the media^b^ in a water bath at around 95°C until complete melting and add the supplements.

a Do not heat the solution.

b Do not use a microwave to melt the media if a magnetic stir bare is in the bottle.

### *Drosophila melanogaster* vials

Fruit flies’ vials are closed with a cotton plug and autoclaved. To avoid watering the cotton during sterilization, they are tightly wrapped in aluminum foil before autoclaving (Figure 1). Fruit flies’ vials are used empty or poured with different media Table 2.

**Table 2:** Vials and media used in the protocol

Name used in the present protocol	Media and volume	Advices and comments
Empty vial	No media.	No condensation due to autoclaving should be present on the wall. Sterilizations must be done in advance.
BHIA_gsy_ vial^a^	2 mL of BHIA_gsy_ (Table 1).	Stored at 4°C.
Starving vial^a^	2 mL of agar (Table 1).	Stored at 4°C.
Infection vial^a^	9 mL of agar (Table 1). A sterile filter is placed on the agar. Sterilize filters in an autoclavable envelope wrapped in aluminum foil. Just before infection use sterile forceps (sterilized in an autoclavable envelope) to place the filter on the surface of the agar.	A volume of 9 mL is chosen for practical reasons: if there is not a sufficient volume of agar, the sterile filter is difficult to place at the surface of the media. This volume is not critical and can be modified by the user.
Fruit fly’ food vial^a^ (modified Bloomington with or without antibiotics)	8 mL of Bloomington food supplemented with 1 mL of sucrose (500 g/L) and antibiotics (Tables 1 and 3).	Bloomington food is autoclaved in the vial and supplemented with sucrose and antibiotics (if needed) after cooling. Steps for the formulation of this media are describe from steps 1–14.

a All media should be at a temperature between 18°C and 25°C when transferring flies.

### Antibiotics cocktail

Antibiotics are used to generate GF fruit flies before infection with the bacteria of interest. Antibiotics presented in Table 3 are used all together to supplement BHIA_gsy_ and modified Bloomington.

***Note:*** Throughout the protocol, when it is mentioned “supplemented with antibiotics” it always refers to the four antibiotics all together at the final concentration presented in Table 3.

**Table 3:** Antibiotics cocktail used in the protocol

Antibiotic	Stock concentration (mg/mL)	Dilution buffer	Final concentration in the media (μg/mL)
Ciprofloxacin^a^	20	Milli-Q water^b^	20
Kanamycin^a^	50	Milli-Q water^b^	100
Ampicillin^a^	100	Milli-Q water^b^	100
Erythromycin^a^	100	95% ethanol	100

a Stored at −20°C, thawed on ice.

b Antibiotic solution filtered through a 0,2 μm filter and maintained sterile.

### Proteinase K (25 μg/mL)

Proteinase K (25 μg/mL) is used in step 49 to perform antigen retrieval and is prepared as presented in the Table 4.

***Note:*** Store aliquots of stock and working solutions of proteinase K at −20°C.

**Table 4:** Preparation of proteinase K

Reagent	Stock concentration	Dilution	Final concentration
Working solution of proteinase K	25 mg/mL, dissolve 100 mg of lyophilized proteinase K in 4 mL of buffer TE-CaCl_2_ (Table 5)	1/1,000 in TE-CaCl_2_ (Table 5).	25 μg/mL

**Table 5:** Composition of TE-CaCl_2_ buffer

Reagent	Final concentration	Stock concentration	Volume
Tris-base	50 mM	1 M Tris-base (12,11 g in 100 mL milli-Q H_2_O)	5 mL
EDTA	1 mM	0,05 M Sodium EDTA (1,86 g in 100 mL milli-Q H_2_O)	2 mL
CaCl_2_	5 mM	0,5 M CaCl_2_ (5,55 g in 100 mL milli-Q H_2_O)	1 mL
Triton X-100	0,5% (v/v)		500 μL
Milli-Q water			To a final volume of 100 mL^a^

a Add 80 mL of milli-Q water, adjust at pH 8 and complete to a final volume of 100 mL with milli-Q water.

## Step-by-step method details

This protocol describes four different steps in order to use *D. melanogaster* as an animal model to study *Propionibacterium* spp. biofilm *in vivo*: (1) generation and maintenance of GF *D. melanogaster*; (2) culture and preparation of *Propionibacterium* spp. biofilms; (3) oral infection of GF fruit flies; and (4) evaluation of the biofilm.

### Germ-free *Drosophila melanogaster* line generation

#### Fly food: modified Bloomington

The Bloomington media is prepared according to the Bloomington *Drosophila* Stock Center instructions (BDSC, 2019) with some modifications in the composition.

1. Heat and homogenize in tap water, one at a time, the ingredients listed in Table 6.

**Table 6:** Initial ingredients to homogenize and boil in tap water to prepare the modified Bloomington food

Reagent	Quantity added to 1 L of tap water^a^	Final concentration^b^
Agar	9 g	8,5‰ (w/v)
Yeast Brewers	80 g	75,7‰ (w/v)
Yeast Extract	20 g	18,9‰ (w/v)
Peptone	20 g	18,9‰ (w/v)
Sucrose	30 g	28,4‰ (w/v)
Glucose	60 g	56,8‰ (w/v)
MgSO_4_	0,5 g	0,5‰ (w/v)
CaCl_2_	0,5 g	0,5‰ (w/v)

a Routinely, this recipe is adjusted for an initial volume of 7 L tap water resulting in 7,4 L of media after the addition of all the ingredients to the water.

b Approximate final concentrations after boiling, evaporation and addition of the last components (p-Hydroxy-benzoic acid methyl ester and propionic acid) from steps 2–5.
2. Stir the mixture until it boils and turn-off the heat source.
3. Let the mixture cool down to 60°C.
4. Add 6 mL (for 1 L of media) of p-Hydroxy-benzoic acid methyl ester diluted in 95% ethanol and stir.

***Note:*** Adjust this volume according to the initial volume of tap water used. The approximate final concentration of p-Hydroxy-benzoic acid methyl ester in the media is 5,7‰ (v/v).

5. Add 10 mL (for 1 L of media) of propionic acid and stir.

***Note:*** Adjust this volume according to the initial volume of tap water used. The approximate final concentration of propionic acid in the media is 9,5‰ (v/v).

6. Distribute manually 8 mL of media per vial and let cool down.
7. Cover vials with plastic wrap and store at 4°C until next step.

**Pause point:** Bloomington vials can be stored for a week at 4°C.

8. Close each vial containing the food with a cotton ball and wrap it tightly with aluminum. Autoclave the vials and let them cool down at graspable temperature.

**CRITICAL:** From this step the user must work under sterile conditions throughout. All manipulations of the food should be performed in a sterile way, working with a Bunsen burner or BSC following good laboratory practices.

9. Mix well by vortexing thoroughly.
10. Supplement the 8 mL of food with 1 mL of sterile sucrose (Table 1).

***Note:*** If antibiotics are needed, they can be supplemented at the same time than the sucrose (Tables 2 and 3).

11. Mix the food with the supplements by pipetting three times up and down.
12. Pipette the entire mixture to a new sterile vial.

***Note:*** This step is to not have condensation and media on the vial wall due to autoclaving and mixing. On step 10 we recommend the use of a 10 mL pipette to add 1 mL sucrose and then to use the same pipette to mix (step 11) and transfer to a new vial (step 12).

13. Close the vial with the cotton ball and aluminum foil. Let it cool down on ice until the food is fully solidified.

***Note:*** It is important to quickly solidify the food to get a homogenous media.

14. Store the vials at 4°C.

#### Generation of germ-free *Drosophila melanogaster*

**Timing: 1.5–2 months**

These steps detail how to develop GF line of *D. melanogaster*. The generation of GF fruit flies is a two months long procedure. Afterwards, the GF line can be maintained and crossed in a sterile way for further experiments.

**CRITICAL:** All fruit flies’ manipulations should be performed in BSC using sterile materials and media. Spray-off all materials with 70% ethanol before starting.

15. Transfer *D. melanogaster* in a starving vial and incubate for 6 h at 25°C and 60% humidity.

**Figure 2:**
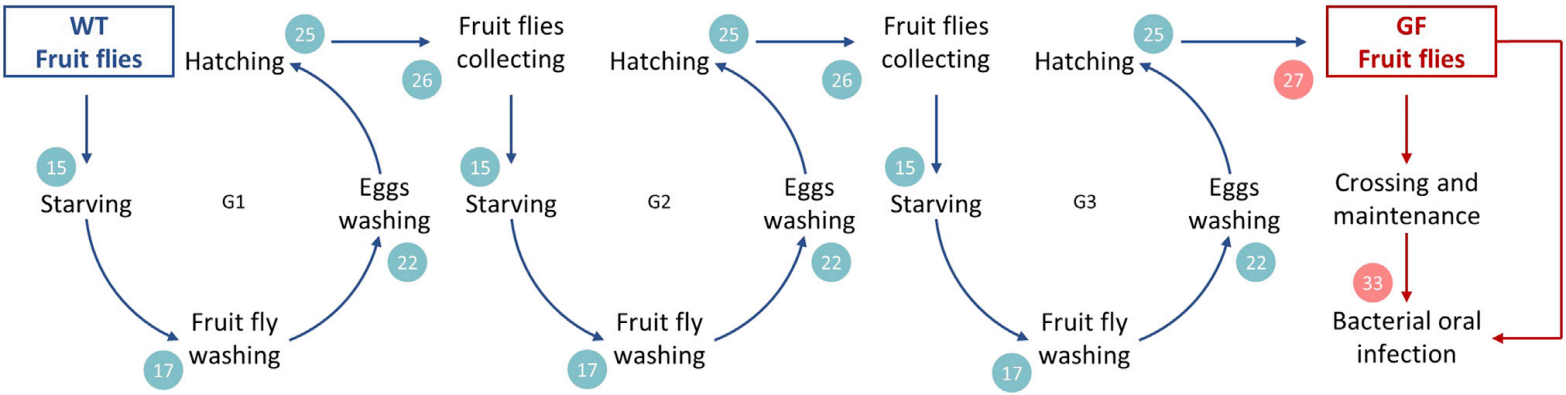
Germ-free *Drosophila melanogaster* line generation GF fruit flies are obtained from WT fruit flies after three generations (G1, G2 and G3) of washing and raising on sterile media with antibiotics (steps 15–26). Blue arrows show steps for the generation of GF fruit flies. Red arrows correspond to GF fruit flies. All steps should be performed in a BSC in sterile conditions.

***Note:*** The procedure (from steps 15–25) is repeated three times in order to generate GF fruit flies. Step 15 initially starts with wild-type (WT) non-sterile *D. melanogaster*, followed two times by the fruit flies generated in step 26 (Figure 2).

***Note:*** Fruit flies’ fertility declines with age, start the experiment with young fruit flies (< 3 days old).

16. After starving, place the vial on ice to anesthetize fruit flies.
17. Wash fruit flies with a sterile washing kit (Figure 1).
  a. Add 2 mL of 70% ethanol to the vial and quickly filter fruit flies through a cell strainer.
  b. Wash by immersing the cell strainer for 2 min in a Petri dish containing 2,7% sodium hypochlorite.
  c. Flush with 70% ethanol.
  d. Immerse the cell strainer with the fruit flies for 10 min in a Petri dish containing autoclaved milli-Q water.
  e. Place the cell strainer on the blotting paper to absorb the excess of water.

***Note:*** Pipette all the liquids with sterile plastic transfer pipettes.

18. Let the fruit flies air dry in the BSC by covering the cell strainer with an empty vial.

***Note:*** Air dry fruit flies until they no longer stick to each other or to surfaces of the cell strainer (approximately 10 min).

***Note:*** after step 17, fruit flies are anesthetized due to all the washing steps.

19. Transfer fruit flies in an empty sterile vial to let them recover (between 15–30 min).
20. Transfer fruit flies into a vial with 2 mL BHIA_gsy_ supplemented with antibiotics.

***Note:*** Transfer fruit flies when they are fully conscious and mobile. If they are still anesthetized during the transfer they will stick to the medium. Place vials sideways to let the fruit flies recover.

21. Incubate for 18–24 h at 25°C and 60% humidity.
22. Collect and dechorionated fruit flies’ eggs with a new washing kit.
  a. Remove fruit flies from the vial.
    ***Note:*** Dispose adults flies following the recommendations presented in the step “Fruit flies disposal”.
  b. Add 2 mL of sterile water.
  c. Gently brush the surface of the media to collect eggs.
  d. Transfer the mixture (water and eggs) into a cell strainer.
  e. Repeat steps 22.a–22.d if there are eggs left on the media surface.
  f. Wash eggs in the cell strainer.
  g. Immerse the cell strainer in a Petri dish containing 2,7% sodium hypochlorite for 2 min.
  h. Wash with 70% ethanol.
  i. Immerse the cell strainer into a Petri dish with water for 10 min.

23. Take out the cell strainer from the water.
24. Transfer washed eggs with a plastic transfer pipette to fresh autoclaved modified Bloomington food supplemented with antibiotics.
25. Incubate the vial at 25°C and 60% humidity until new fruit flies hatch.

***Note:*** Depending on the environmental conditions and quality of the fruit flies, the duration of life cycle of fruit fly can vary. A delay in fly growth and size has been noticed with GF fruit flies. In the conditions tested larva should start moving and growing on the media 2–3 days after eggs collection. Fruit flies start hatching after about 15 days.

26. Repeat two more-time steps 15–25 starting with the newly hatched fruit flies from step 25 (Figure 2).

***Note:*** When pupa start to hatch, collect young fruit flies each day into a BHIA_gsy_ vial supplemented with antibiotics. Collect the fruit flies for three days before repeating the step 15.

27. After three generations (G1, G2, G3; Figure 2) fruit flies are considered sterile and can be used as an *in vivo* model.

***Note:*** Validation of the germ-free status can be performed both with culturing and molecular methods and is documented in our recent publication (Bronnec and Alexeyev, 2021).

**Pause point:** After the third generation, GF fruit flies can be maintained and crossed on sterile food with antibiotics until the desired number of young GF fruit flies is reached (Figure 2; troubleshooting 2).

### *Propionibacterium* spp. biofilm preparation

**Timing: 11 days**

In natural environment, biofilm dispersion also known as “seeding dispersal” is the last step of the biofilm formation facilitating the colonization of new sites and the development of a new biofilm (Rumbaugh and Sauer, 2020). In this protocol, sessile *Propionibacterium* spp. are used to infect GF fruit flies instead of planktonic cells in order to enhance the biofilm formation inside their gut.

***Note:*** This protocol has been optimized to develop mono-species biofilm of *Propionibacterium* spp.

***Note:****Propionibacterium* spp. cultures are performed in anaerobic environment. Plates and flasks are incubated in jars with an anaerobic atmosphere generation sachet. Planktonic cultures are performed in a 10 mL tube fully filled and closed. All incubations are performed in a laboratory incubator at 37°C with an agitation of 250 rpm for planktonic cultures.

**CRITICAL:** The user must work under sterile conditions throughout following usual good microbiological laboratories practices. Use a Bunsen burner or BSC when working with *Propionibacterium* spp. (troubleshooting 3).

28. Plating *Propionibacterium* spp.
  a. Slightly scratch the surface of frozen *Propionibacterium* spp. glycerol stock with a sterile inoculating loop.
  b. Streak the loop across a blood agar plate.
  c. Invert and incubate the plates at 37°C for 72 h anaerobically.
29. Preculture of *Propionibacterium* spp.
  a. Pick-up one colony from the plate from step 28.c with a sterile inoculating loop and inoculate 10 mL of BHI_g_.
  b. Incubate at 37°C with 250 rpm agitation for 48 h anaerobically.
30. Culture of *Propionibacterium* spp.
  a. Inoculated 9,5 mL of BHI_g_ in a T-25 cell culture flask with 500 μL of the preculture from step 29.b.
  b. Incubate the flask horizontally at 37°C for 6 days anaerobically with a medium change every 48 h.

**Figure 3:**
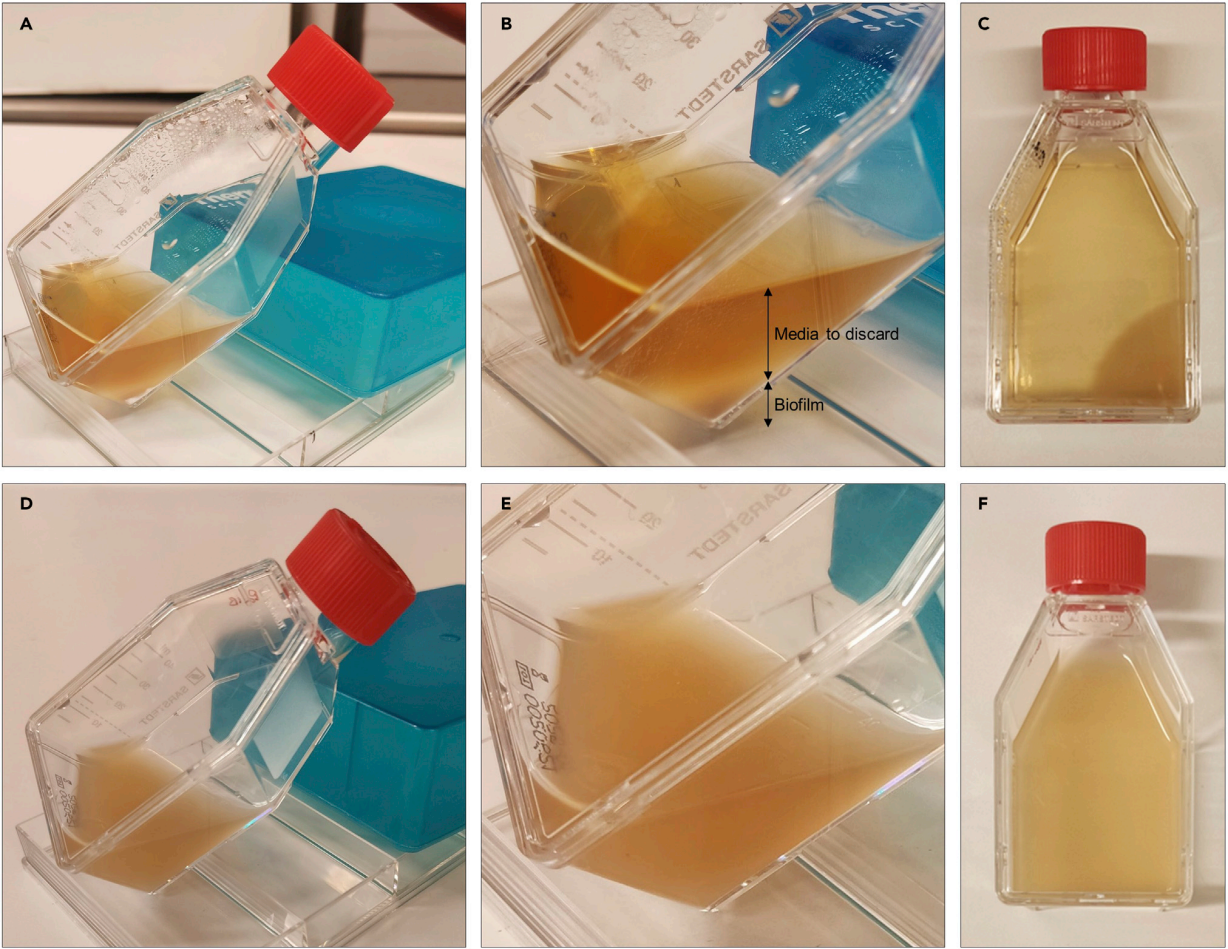
Biofilm of *Propionibacterium* spp in T-25 cell culture flask To let the biofilm pelleted at the corner of the flask by gravity (to change the media or to recover the biofilm), the flask is tilted for about 10 min between 18°C and 25°C. (A and D) An in-house-built system is used to create an angle (a square tissue culture dish and a lid of a pipet tips box). (B and E) close-up of (A) and (D) respectively. (B) The biofilm is pelleted at the corner of the flak and the media should appear clear. (C) After media changes (about 8 mL removed and 10 mL BHI_g_ added) the biofilm is visible at the corner of the flask. (D–F) When the culture is contaminated or if the culture has been shaken, the media appears turbid and no biofilm is visible.

***Note:*** To change the media, tilt the flask (± 45° angle) for about 10 min to let the biofilm pelleted at the corner of the flask by gravity (Figures 3A and 3B). Without disturbing the pellet (lowest speed of the Pipetboy), carefully pipette the supernatant (about 8 mL) and discard it. Add slowly 10 mL of BHI_g_.

### Oral infection of *Drosophila melanogaster*

**Timing: 10 days**

31. Preparation of fruit flies before infection.
  a. Use newly born to 3-days-old GF fruit flies from the step 27.
    ***Note:*** The population density is approximately 30 fruit flies per vials.
  b. Transfer fruit flies two times for 24 h on BHIA_gsy_ supplemented with antibiotics and incubate at 25°C and 60% humidity.
  c. Transfer fruit flies on BHIA_gsy_ without antibiotics and incubate for 24 h.

32. Recover the 6-days old biofilm of *Propionibacterium* spp. formed at the bottom of the T-25 culture flask (Figure 3B; troubleshooting 4).
  a. Tilt the flask (± 45° angle) for about 10 min to let the biofilm pelleted by gravity.
  b. Pipet slowly 1 mL of the biofilm at the corner of the flask and transfer it to a sterile 1,5 mL micro-tube.
  c. Centrifuge for 3 min at 1,150 g between 18°C and 25°C.
  d. Discard the supernatant by pipetting and resuspend the pellet in 100 μL of BHI_s_ by pipetting up and down.
  e. Pipette 100 μL of the concentrated biofilm suspension on the filter of an infection vial.
  f. Let the filter dry/absorb for 30 min.
33. Transfer fruit flies from step 31.c and incubate for 24 h (troubleshooting 5).
34. Repeat the infection procedure (step 32) using the fruit flies from step 33 every 24 h for 6 days.
35. After 6 days of infection, transfer the fruit flies on BHIA_gsy_ without antibiotics and incubate for 24 h.

***Note:*** This step is to let fruit flies to shed unattached bacteria.

### Formalin-fixed paraffin embedded tissue samples and sectioning

**Timing: 3–4 days**

36. Fruit flies sacrifice.
  a. Anesthetize fruit flies on ice.
  b. Place them into a fine pore tissue cassette using forceps.
  c. Place the small cassette in a larger tissue embedding cassette.

***Note:*** Grab the fruit flies wings to not damage their abdomen.

***Note:*** Label the tissue embedding cassette on the angular writing area as well as the side of the cassette with a pencil. Markers are erased with solvents during formalin fixed paraffin embedded (FFPE) procedure.

**CRITICAL:** From steps 37–40 the work must be done under chemical fume hood, the user must wear personal protections and follow his laboratory procedures and guidelines when working with hazardous chemicals. The user should consult product safety data sheet for the chemical substances used.

***Note:*** FFPE steps are performed between 18°C and 25°C. Fruit flies are placed in a tissue embedding cassette to keep them submerged in liquids during all the procedure. Liquid solutions are in specimen containers with a volume of 50 mL of liquids for maximum five embedding cassettes. Tighten the lid to close the container after immersion of the cassette.

37. Immerse the cassette in buffered formalin (or fixative alternative) in a specimen container for at least 12 h (long enough for it to penetrate through every part of the specimen).
38. Rinse two times 10 min with PBS 1X.

***Note:*** PBS 1X is prepared by diluting 10 times PBS 10X (CSHProtocols, 2007) in sterile milli-Q water.

39. Dehydrate with serial baths of ethanol by transferring tissue embedding cassettes from one container to another:
  a. Two baths of 1 h each with 70% ethanol.
  b. One bath of 1 h with 80% ethanol.
  c. One bath of 1 h with 90% ethanol.
  d. Three baths of 1,5 h each with 100% ethanol.

***Note:*** Before dehydration steps, dilute absolute ethanol in sterile milli-Q water to freshly prepare 70%, 80% and 90% ethanol solutions.

40. Perform tissue clearing with 3 baths of Xylene for 1,5 h each.

***Note:*** Substitutes are available as an alternative to Xylene but we did not test these clearing agents: HistoChoice® Clearing Agent (Sigma Aldrich, Cat#H2779) or Xylene Substitute (Sigma Aldrich, Cat#A5597).

41. To infiltrate the tissue with paraffin, immerse the sample two times 2 h in paraffin wax for tissue embedding (58°C–60°C) and cast the sample in paraffin blocks stainless steel embedding molds.

**Figure 4:**
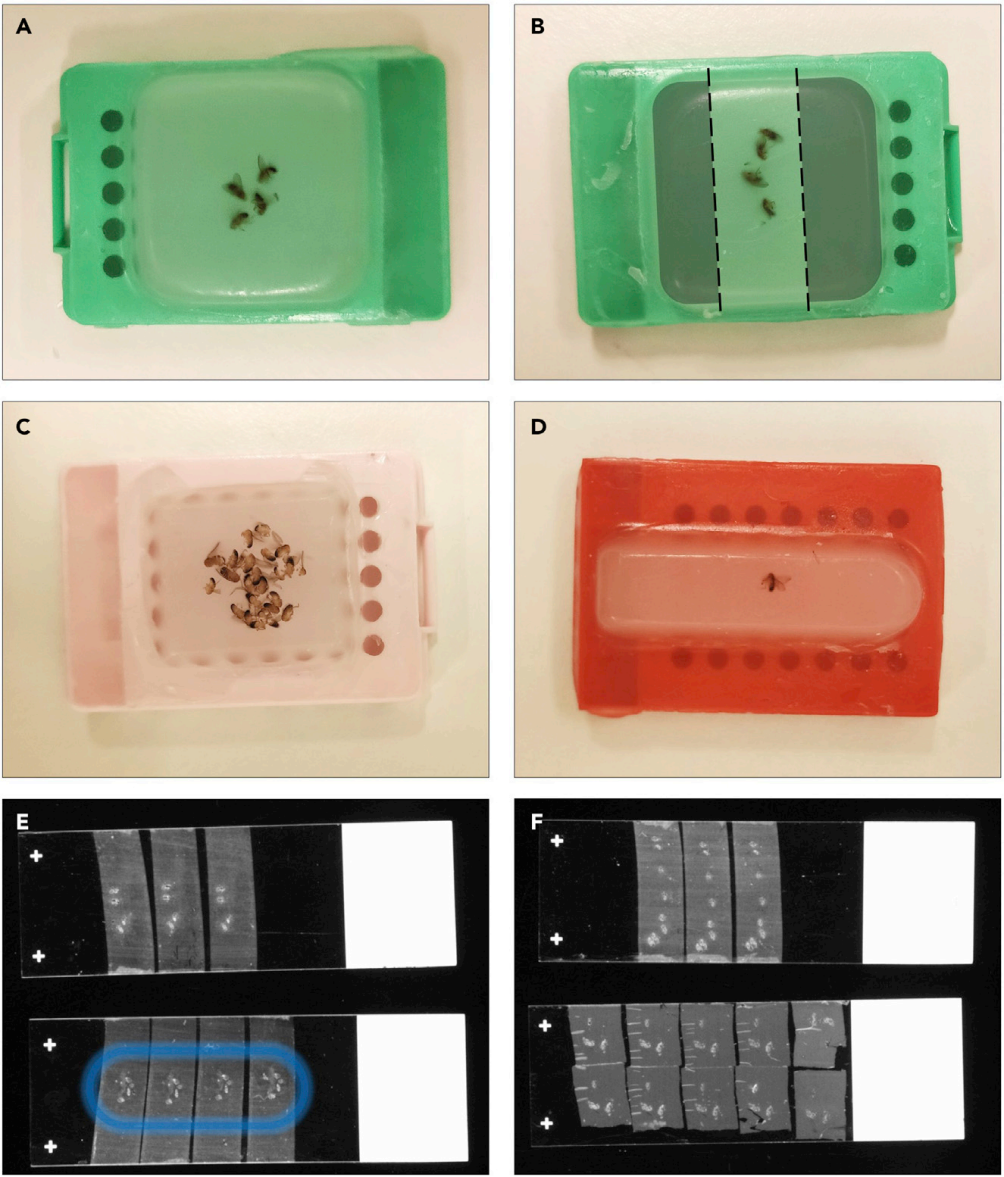
Formalin-Fixed Paraffin-Embedded blocks and sections (A and B) Correct positioning of fruit flies in the paraffin block. The gray area (B) shows the excess of paraffin to trim with a scalpel before sectioning in order to place more sections on one slide. (C) A paraffin block with too many fruit flies, some appears damaged. (D) Fruit fly embedded in a rectangular mold (less paraffin to trim). (E) Correct placement of sections on a slide to be processed for immunofluorescence staining. The blue circle on the bottom slide shows how the hydrophilic border can be drawn after deparaffinization. During immunostaining, solution (steps 49–51, 53, 55 and 56) should be added within the circle. (F) Placement suitable for visualization without processing but fruit flies are too close to the edge of the slide to draw a hydrophilic border for a staining procedure.

***Note:*** The infection experiment starts with 30 fruit flies per vial. During the infection procedure, some will die. The user should determine how many blocks to prepare according to his scientific question and the number of replicates wanted. We recommend a number of 4–6 flies per paraffin block, a low number is easier to cast in paraffin and to process (Figure 4). Orientation of fruit flies in the block is not critical.

**Pause point:** After preparation, FFPE blocks are stored at 4°C (alternatively they can be stored between 18°C and 25°C).

42. Cut the FFPE blocks at 4 μm using a microtome. Follow a general microtome cutting procedure.

***Note:*** Paraffin blocks should always be cold before cutting (stored at 4°C or chilled on a cold surface).

***Note:*** Use high quality, disposable and sharp blades.

43. Transfer each section to a 45°C water bath using fine forceps.

***Note:*** Use clean milli-Q water in the flotation bath and dislodged air bubbles with a painting brush before sectioning.

44. Pick-up floating section with a slide. Use highly adhesive slides for immunofluorescence or regular slides if unprocessed visualization is planned.

***Note:*** Pick-up the section immediately, do not let it stand on the water bath, the sample cracks easily.

***Note:*** If needed several sections can be placed on one slide if excess of paraffin is trimmed (Figures 4B, 4E, and 4F).

45. Drain the water with a tissue and let the slides dry vertically in a staining rack between 18°C and 25°C for at least 12 h.

***Note:*** The user should label the slides with a pencil. Several immersion steps are performed and permanent markers are erased with solvents.

### *In vivo* biofilm visualization

In the present protocol, two methods of visualization are presented: (i) deparaffinization and immunostaining to visualize specifically *Propionibacterium* spp., (ii) unprocessed slides observation to evaluate biofilm distribution.

#### Specific observation using immunolabeling

**Timing: 2 days**

46. Deparaffinization and rehydration of sections.
  a. Place the slides from step 45 on a slide staining rack.
  b. Melt the paraffin by incubating the slides at 60°C for 1 h.
    ***Note:*** All the baths for slides described hereafter are performed by immersing the entire rack in staining jars containing the different solutions.
    **CRITICAL:** Samples detach easily from the slide. After the paraffin is melted, baths for deparaffinization and rehydration should be gentle. Do not shake the rack in the solution nor tap it on the edge of the container to remove excess liquids (troubleshooting 6).
  c. Immerse the slides two times for 10 min in xylene to deparaffinize.
  d. Rehydrate by immersing the slides in the following solutions:
    i. 100% ethanol 2 min.
    ii. 70% ethanol 2 min.
    iii. 30% ethanol 2 min.
47. Wash the slides by immersing the rack two times 5 min in PBS 1X.
48. Draw a circle with a hydrophobic barrier pen around slide-mounted tissue to create a water-repellent barrier (Figure 4E).

***Note:*** On steps 49–51, 53, 55 and 56 the volume of the drop added to the tissue depends on the initial repartition of the flies in the paraffin block and the size of the circle draw in step 48 with the hydrophobic barrier pen. A good placement of the fruit flies in the paraffin block is presented in Figure 4.

***Note:*** During incubation steps (49–51, 53, 55 and 56), slides are placed horizontally in a moist chamber (cover the bottom with a thin layer of distilled water).

**CRITICAL:** Do not add the drop directly on fruit flies’ tissues to not damage the sample. Apply the drop on the glass and let it gently cover the tissues.

**CRITICAL:** Between each incubation steps (49–55), gently tap the edge of the slide against a paper towel to remove excess reagent before adding the next one.

49. Perform antigen retrieval by treating the hydrated tissue sections with proteinase K (25 μg/mL) for 15 min at 37°C.
50. Add normal horse serum blocking solution and incubate 20 min between 18°C and 25°C.
51. Apply the primary antibody on the fruit flies’ tissues and incubate for 1 h between 18°C and 25°C.

***Note:*** In the protocol herein, we use the following antibodies depending on the species used to infect the GF fruit flies: mouse monoclonal anti-*P. acnes*, chicken polyclonal anti-*P. granulosum*, rabbit polyclonal anti-*P. avidum*.

***Note:*** Antibodies dilutions are performed in normal horse serum blocking solution and used at 1/400, 1/10,000 and undiluted for anti-*P. granulosum*, anti-*P. avidum* and anti-*P. acnes* antibodies respectively.

52. Wash the slides by immersing the rack two times 5 min in PBS 1X.
53. Apply the secondary antibody and incubate for 1 h between 18°C and 25°C in the moist chamber and protect from light.

***Note:*** The conjugate used on step 53 depend on the primary antibody used in step 51. They are labeled with Alexa Fluor 555 (orange fluorescence), Alexa Fluor 488 (green fluorescence) or Alexa Fluor 647 (red fluorescence).

***Note:*** Secondary antibodies are diluted in normal horse serum blocking solution according to the manufacturer’s instructions.

54. Wash the slides by immersing the rack two times 5 min in PBS 1X.
55. Add a drop of DAPI (4 μg/mL) on fruit flies’ tissues and incubate for 2 min.
56. Add drop of glycerol/PBS antifade.
57. Cover the sample with a coverslip starting from one side of the sample and drop it quickly on the sample to avoid air bubbles.
58. Seal the edges with a coverslip sealant.
59. Store the slides in darkness (to avoid photobleaching) and at 4°C.
60. Take image of the slides as soon as possible not to lose the quality of fluorescence.

***Note:*** Slides are usually stable for several weeks.

61. Capture images using an epifluorescence or confocal microscope using appropriate wavelength.

***Note:*** DAPI and secondary antibodies with Alexa Fluor 488, 555, 647 have been use to labeled the samples*.* Immunostained sections were analyzed on a Zeiss Axio Imager M2 microscope using 10×/0.3, 20×/0.8 and 63×/1.4 oil objectives. Images were captured with a Hamamatsu ORCA-Flash 4.0 LT digital CMOS camera. Visualization of DAPI, Alexa Fluor 488, 555 and 647 was achieved simultaneously in each slide by using Zeiss Filter Set 01 (ex: BP 365/12, em: LP39738), HE (Ex: 470/40, Em: 525/50), 43 (Ex: 545/25, Em: 605/70) and 50 (Ex: 640/30, Em: 690/50). Image processing was performed using the ZEN software (Carl Zeiss Vision, Germany).

#### Unprocessed slides observation

**Timing: 1–2 days**

This step can be performed when the procedure has been validated with immunofluorescence first. With unprocessed slides the biofilm appears as a large compact structure. The specificity of the bacteria present in the biofilm should be initially confirmed with immunofluorescence.

62. Slides from the step 58 can be directly observed without deparaffinization and without staining using a light microscope.

## Expected outcomes

The aim our protocol was to provide to users an easy to carry out method to (i) generate GF fruit flies, (ii) to infect them with a mono-species biofilm of *Propionibacterium* spp. and (iii) to visualize the biofilm in the gut of the fruit flies.

Using the method, we have developed, we were able to generate GF fruit flies. This status was confirmed with both molecular and culture-based methods in our recent publication (Bronnec and Alexeyev, 2021). Using 16S rRNA PCR analysis and microbial culture from crushed fruit flies or their excretions, no DNA amplification or bacterial growing were observed (Bronnec and Alexeyev, 2021).

**Figure 5:**
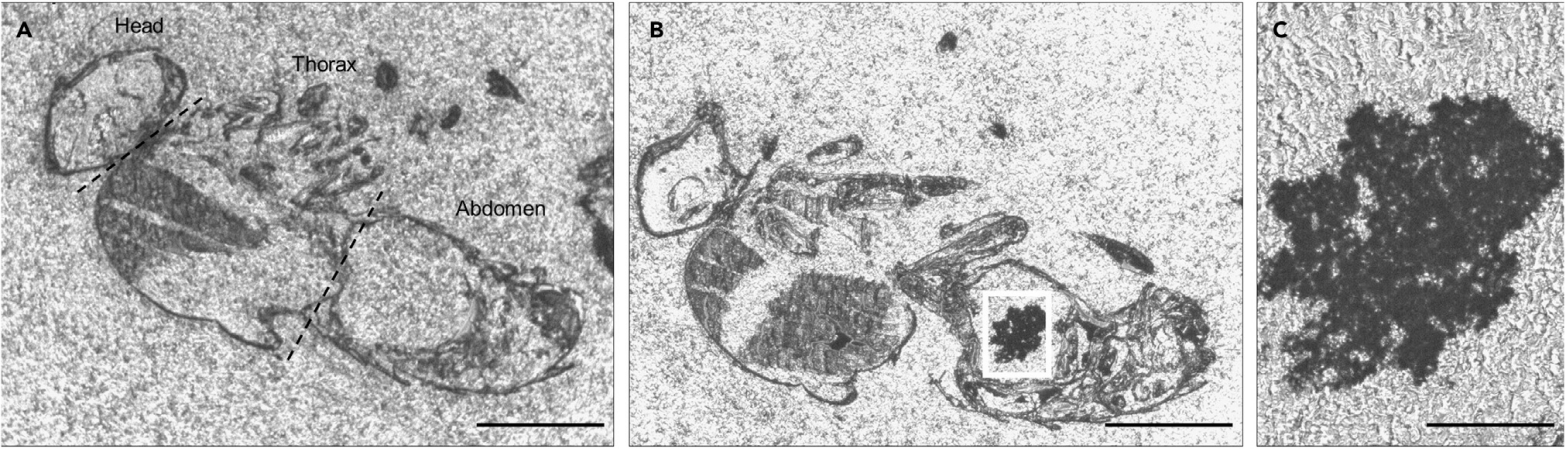
Bright field observation of unprocessed section of a fruit fly (A) 4 μm section of a fruit fly without biofilm. Fruit fly body is divided into three anatomical parts: head, thorax, and abdomen. (B) Fruit fly infected with *P. acnes* biofilm. (C) Close-up of figure A. Scale bar: 500 μm.

**Figure 6:**
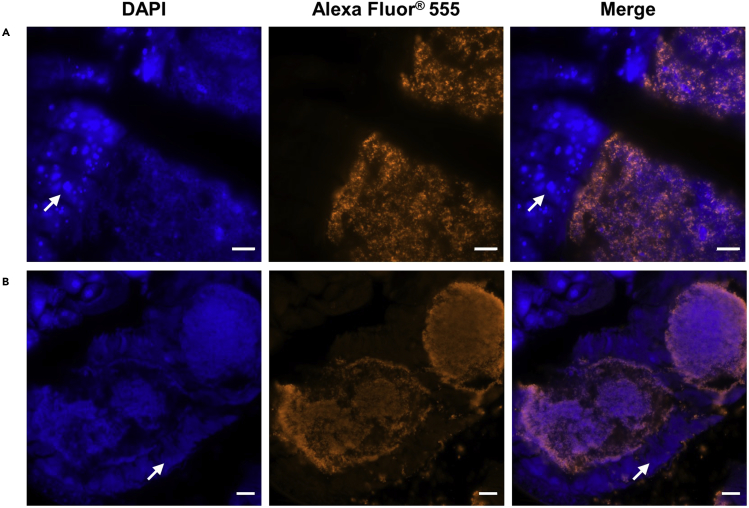
Immunofluorescence of *in vivo Propionibacterium acnes* biofilm (A and B) *P. acnes* is labeled with anti-*P. acnes* monoclonal antibody/Alexa Fluor® 555 goat anti-mouse IgG. Host and bacterial nuclei are stained with DAPI. Arrows highlight the fruit fly gut wall. Scale bar: 20 μm.

**Figure 7:**
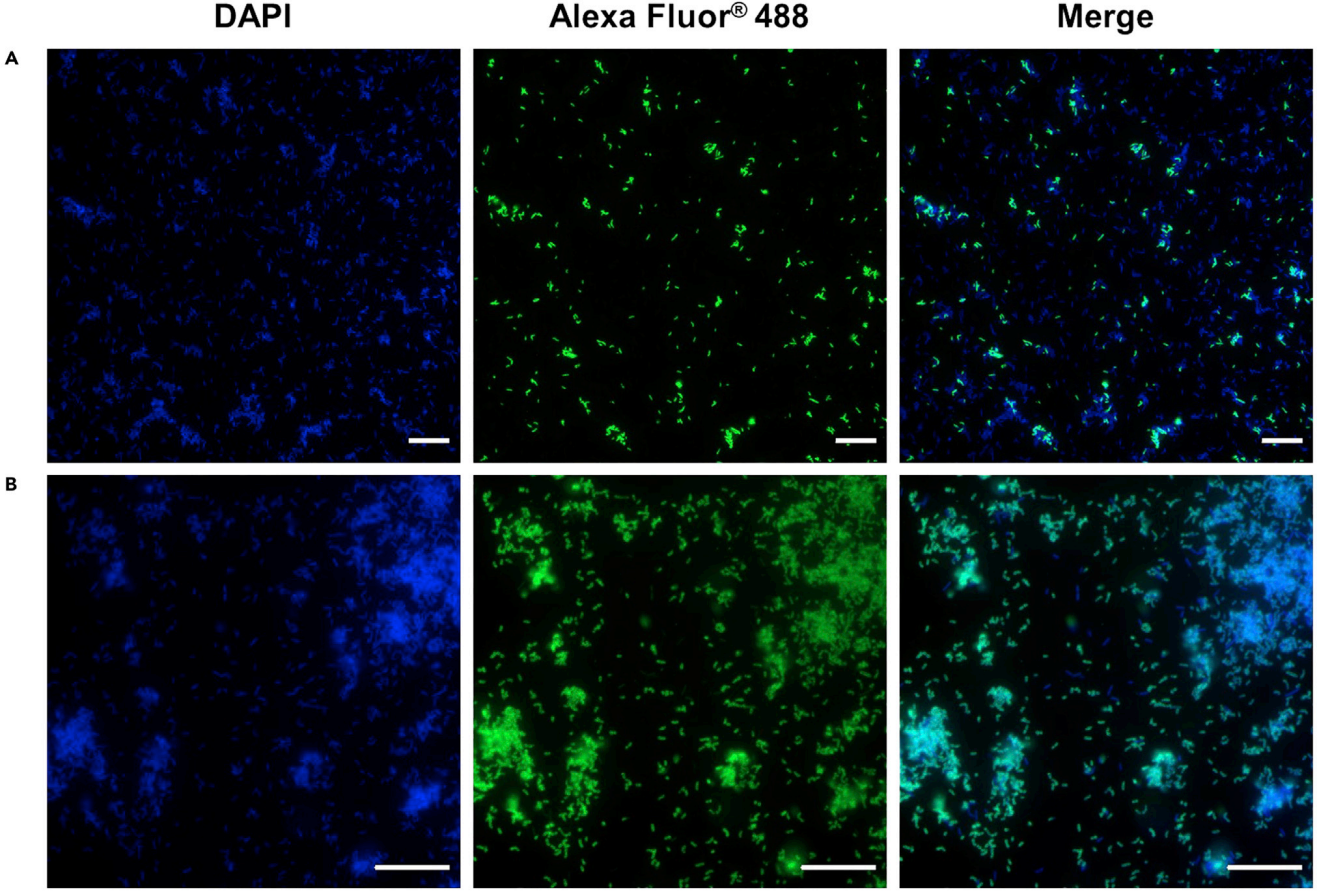
Immunofluorescence of a non-germ-free *Drosophila melanogaster* infected for three days with *Propionibacterium acnes* (A and B) Sections of (A) WT and (B) contaminated GF fruit flies are stained with DAPI and anti-*P. acnes* monoclonal antibody/Alexa Fluor® 488 goat anti-mouse IgG. The presence of rod-shaped bacteria other than *P. acnes* is visible in the merge image. Scale bar: 20 μm.

The feeding infection method leads to the fruit fly gut colonization and the development of a biofilm attached to the epithelial cells of the gut. These outcomes were demonstrated by microscopy. Unprocessed fruit fly sections were visualized with a bright field microscope (Figure 5). Biofilms were identified as large black and compact aggregates in the fruit fly abdominal area. To confirm that the black structures observed in the bright field, correspond to *Propionibacterium* spp., immunofluorescence assay with specific antibodies were used showing *P. acnes* biofilm inside the fruit fly (Figure 6). During the immunostaining procedure the use of DAPI (to stain prokaryotic and eukaryotic nuclei) helps to identify the presence of contaminations. In the merge image in Figure 7, bacteria identified with DAPI but not labeled with Alexa Fluor® 488 correspond to a contamination. Using scanning electron microscopy, we have observed the samples with high resolution to confirm that *Propionibacterium* was organized as a biofilm by visualizing the matrix structure (Bronnec and Alexeyev, 2021).

As described in the publications, Bronnec and Alexeyev (2021) and Bronnec et al. (2022), unprocessed slides visualization was the main method used to evaluate the biofilm formation. In the studies, the entire paraffin block was cut and each section was observed to evaluate the biofilm distribution. Using in house defined criteria (e.g., biofilm positive sections were section with a large black and compact structures completely filling the lumen; Bronnec and Alexeyev, 2021), we were able to evaluate the biofilm distribution in the gut and the efficacy of biofilm dispersing enzymes: The results are not presented in the present protocol but available in our publications (Bronnec and Alexeyev, 2021; Bronnec *et al.*, 2022).

This *in vivo* model provides now the opportunity to develop bacterial biofilms in anaerobic environment and attached to epithelial cells. The feeding procedure can be used to modify the biofilm environment (fruit fly diet supplemented with lipids for example) or to treat the biofilm with components such as biofilm dispersing enzymes (Bronnec and Alexeyev, 2021; Bronnec *et al.*, 2022).

## Limitations

The protocol herein is limited to GF fruit flies generation, infection with *Propionibacterium* spp. and biofilm visualization. This *in vivo* model has been used in our recent publications where more informations are provided regarding the outcomes validation such as the method to validate the germ-free status, the visualization of the biofilm matrix using scanning electron microscopy and the application of the *in vivo* model to test biofilm dispersing drugs (Bronnec and Alexeyev, 2021; Bronnec *et al.*, 2022).

This protocol has been validated with three species of *Propionibacterium* (*P. acnes*, *P. granulosum* and *P. avidum*). So far, no data have been generated for other bacterial species. The viability of this model will depend on the pathogenicity of the bacteria and the production of virulence factors that might kill the fruit fly.

The ability of *Propionibacterium* spp. to grow and survive at 25°C has been validated (data not presented). All the experiments were consequently performed at this optimal temperature for the fruit flies. Other temperatures of incubation have not been tested.

Work is performed in a BSC, individual sorting of fruit flies (for gender selection for example) is not possible.

## Troubleshooting

### Problem 1

Step “Fly transfer between vials”. Fruit flies stick in the media and die after transfer between vials.

### Potential solution

Recipes and methods proposed in this protocol have been optimized in house. The quality/humidity of the Bloomington food may vary depending on the batches regarding the volume prepared and evaporation during the boiling step. Other solutions are based on defined laboratory culture media and their preparation is straightforward following this protocol.

Before transfer, all the vials should be between 18°C and 25°C as the cold anesthetize fruit flies.

Vial walls should be dry before fly transfer. Open the vial in the BSC to evacuate condensation if needed.

### Problem 2

Step 27. After too long periods on antibiotics supplementation, fruit flies lose their fertility. After several generations, the number of sterile fruit flies available for infection experiment may decrease.

### Potential solution

A transfer of GF fruit flies to a sterile Bloomington without antibiotics is necessary to increase the population of fruit flies. However, infection experiments with *Propionibacterium* spp. (step 31) should always be carried out on flies born in a media containing antibiotics.

### Problem 3

Steps 28–30. Contamination of *Propionibacterium* spp. cultures.

### Potential solution

Sterile conditions should be maintained during bacterial manipulations. Good microbiological practices should be followed using sterile materials and media.

### Problem 4

Step 32. No biofilm formation during *Propionibacterium* spp. cultivation.

### Potential solution

It is critical to follow the procedure presented in this protocol for culturing *Propionibacterium* spp. Flasks should be incubated horizontally, in anaerobiosis and without agitation during the incubation time. Pipetting should be gentle and slow without disturbing the biofilm.

After tilting the flask, as presented in Figure 3, if two phases are not visible and the upper phase appears totally turbid, the culture might be contaminated with other bacteria.

### Problem 5

Step 33: Fruit flies stick to the bacterial culture on the filter during infection.

### Potential solution

Infection is performed with 100 μL of concentered bacterial biofilm in step 32.e. The volume should be spread all over the filter to avoid a wet area in the middle of the filter. An incubation time of 30 min between 18°C and 25°C is necessary before the fruit fly transfer.

### Problem 6

Steps 46–54. Fly section detachment during sample processing.

### Potential solution

The use of highly adhesive slides is recommended (step 44) in order to attached tissue sections firmly to the surface of the slide. Deparaffinization steps should be as gentle as possible.

## Resource availability

### Lead contact

Further information and requests for resources and reagents should be directed to and will be fulfilled by the lead contact, Vicky Bronnec (vicky.bronnec@umu.se).

### Materials availability

Germ-free *D. melanogaster* generated are not available. This protocol is described in order to generate and maintain in house sterile line of fruit flies.

## Data Availability

The published article includes all datasets and codes generated or analyzed during this study.
